# Embodied Information in Cognitive Tasks: Haptic Weight Sensations Affect Task Performance and Processing Style

**DOI:** 10.5709/acp-0172-0

**Published:** 2015-09-30

**Authors:** Kai Kaspar, Alina Vennekötter

**Affiliations:** 1 Department of Psychology, University of Cologne, Germany; 2 Institute of Psychology, University of Osnabrück, Germany

**Keywords:** embodied cognition, weight sensations, cognitive task, task performance, response heuristic

## Abstract

Research in the field of embodied cognition showed that incidental weight sensations influence peoples’ judgments about a variety of issues and objects. Most studies found that heaviness compared to lightness increases the perception of importance, seriousness, and potency. In two experiments, we broadened this scope by investigating the impact of weight sensations on cognitive performance. In Experiment 1, we found that the performance in an anagram task was reduced when participants held a heavy versus a light clipboard in their hands. Reduced performance was accompanied by an increase in the perceived effort. In Experiment 2, a heavy clipboard elicited a specific response heuristic in a two-alternative forced-choice task. Participants showed a significant right side bias when holding a heavy clipboard in their hands. After the task, participants in the heavy clipboard condition reported to be more frustrated than participants in the light clipboard condition. In both experiments, we did not find evidence for mediated effects that had been proposed by previous literature. Overall, the results indicate that weight effects go beyond judgment formation and highlight new avenues for future research.

## Introduction

Currently, one of the most exciting ideas in cognitive science is that parts of our cognition are embodied (Wilson & Golonka, 2013). In this sense, cognition is not an exclusive assignment of the brain but also deeply rooted in the body’s interaction with the physical world (e.g., Barsalou, 2008; Kaspar, König, Schwandt, & König, 2014; Wilson, 2002). Current research in this field is dominated by the prevailing focus on how bodily sensations affect higher cognitive functions. Indeed, a bulk of empirical evidence supports the idea of a link between bodily experiences and higher cognitive processes (for current reviews see Lee & Schwarz, 2014; Williams, Huang, & Bargh, 2009). In this context, a substantial research line addresses the interplay between incidental weight sensations and higher cognitions, particularly judgment formation, in various settings. Weight is an influential concept we get in touch with early in life. Especially with the limited physical strength of a child it takes more effort to move heavy things compared to light ones, and to be hit by something heavy hurts more than to be hit by something light (Jostmann, Lakens, & Schubert, 2009). Weight is also a common motive in metaphors; that is, metaphors such as *the gravity of the situation*, *to have weight on your shoulders*, or a *weighty matter* address the concepts of seriousness, potency, and importance. Such metaphors sometimes indicate established functional relationships between certain bodily sensations and higher abstract cognitive processes (Kaspar, 2013a). Meier, Schnall, Schwarz, and Bargh (2012) pointed out that “embodied processes have often been identified by the examination of common metaphors in which abstract target concepts are described using concrete source concepts derived from perceptual experience” (p. 706). According to a developmental perspective on cognition, Williams et al. (2009) suggested that abstract concepts, such as importance or seriousness, are difficult to process and understand for the developing brain of a child. But more concrete concepts such as weight are easy to conceptualize because the child physically experiences them while interacting with the environment. Therefore, such concrete concepts provide a basis or “scaffold” in which features of new abstract concepts are integrated. As a consequence, the early sensorimotor childhood experience of physical weight might influence our adult higher cognitive processes like thinking about an issue’s importance without us noticing. Due to this developmental process, we associate physical weight with more abstract but conceptually related cognitions (see also Ackerman, Nocera, & Bargh, 2010; Kaspar, 2013a). This established connection is expressed, for example, in the German language as the German word for *heavy* (*schwer*) is often used synonymously for the word *difficult* (*schwierig*), and the word for *easy* (*leicht*) is the same as for *light*. The mediating role of linguistic correspondences in the context of embodiment phenomena has been emphasized several times (e.g., Ackerman et al., 2010; Jostmann et al., 2009; Kaspar, 2013a). Also, it is the core aspect of the conceptual metaphor theory (Lakoff & Johnson, 1980), according to which abstract concepts are represented by bodily metaphors in a conceptual system. Empirical evidence reliably supports this notion. Altogether, study results suggest that weight not just makes people invest more physical effort in dealing with concrete objects but that weight also significantly affects abstract cognitions—mainly the evaluation of issues and objects on dimensions that are conceptually related to weight (e.g., Ackerman et al., 2010; Chandler, Reinhard, & Schwarz, 2012; Jostmann et al., 2009; Kaspar, 2013a).

With the present work, we broaden the scope to potential weight effects on cognitive performance. We assumed that the impact of basal sensorimotor influences on higher cognitive processes goes beyond the formation of judgments that have been primarily addressed so far. Instead, bodily experiences were expected to influence cognitive performance as well. As Brińol and Petty (2008) stated, “One of the most fundamental things that the body can do” is to affect “the amount of thinking in which people engage when making a social judgment” (p. 188). Thereby, most researchers seem to agree that this effect is most likely to occur when the amount of thinking is not completely constrained by other non-bodily (i.e., disembodied) variables—that is, when the situation is characterized by considerable unfamiliarity or uncertainty (cf. Binder & Desai, 2011; Chandler et al., 2012). In two experiments, we made a first attempt to test whether incidental haptic weight sensations modulate one’s performance in cognitive tasks which were unfamiliar to participants and characterized by some situational uncertainty. In Experiment 1, participants performed an anagram task that provided several degrees of freedom on how to process the test items. We tested whether weight sensations are considered in this context and influence creative thinking. In Experiment 2, participants performed a two-alternative forced-choice task that required an analytic processing style to achieve a fair result. The task was characterized by a high uncertainty regarding the right choice. The two experiments were intended to check whether weight sensations have a differential impact on task processing, depending on the context. Therefore, and in accordance with previous studies (e.g., Ackerman et al., 2010; Jostmann et al., 2009; Kaspar, 2013a; Kaspar & Krull, 2013), physical weight was manipulated by means of a light or heavy clipboard, respectively, held by participants during task processing. Hence, the present two experiments address what Meier at al. (2012) formulated as a research agenda for future studies in the field of embodied cognition: “Future researchers should engage in a phenomenon-based approach, highlight the theoretical boundary conditions and mediators involved, explore novel action-relevant outcome measures, and address the role of individual differences broadly defined” (p. 705).

## Experiment 1

In Experiment 1, we focused on creative thinking and selected an anagram task that is usually considered as an indicator for creativity (e.g., Kumar & Kumari, 1988), cognitive flexibility (e.g., Beversdorf, Hughes, Steinberg, Lewis, & Heilman, 1999), and persistence (e.g., Eisenberger, Kuhlman, & Cotterell, 1992). Anagrams provide some degrees of freedom on how to process such items; that is, they cannot be solved by simply performing an established and stable cognitive routine. Hence, we expected that participants who process an anagram task are (partially) susceptible to embodied information that may interfere with task processing and cognitive effort. Brińol and Petty (2008) stated that influences of embodied informational cues are more likely to occur when thinking is free to vary in different directions. This assumption is supported by a recent study showing that performance in an anagram task is sensitive to prior hand washing (Kaspar, 2013b). Moreover, neurophysiological findings suggest that bodily sensations are more influential when a task requires deeper processing or when the context is less familiar (Binder & Desai, 2011). To meet this precondition, we tested only subjects who were unfamiliar with anagram tasks.

**Figure 1.**
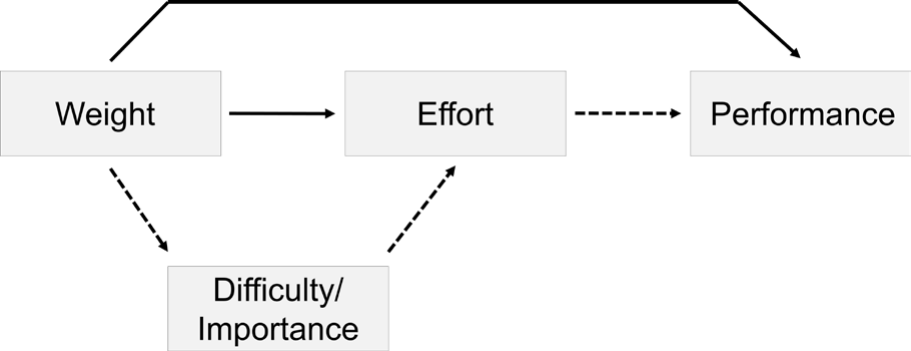
The mediation model of weight effects on task performance that was tested in Experiment 1. Dotted lines indicate the mediated pathways, solid lines the direct pathways.

Jostmann et al. (2009) showed that heaviness sensations are associated with greater investment of cognitive effort triggering higher cognitive elaboration of social issues. According to the authors, in early childhood we learn that dealing with heavy versus light objects generally requires more effort in terms of physical strength or cognitive planning. Thus, we may also associate the experience of weight with the increased expenditure of bodily or mental effort later in life. Consequently, we asked whether the sensation of weight actually increases perceived effort when solving an anagram task. However, while Jostmann et al. (2009) outlined a direct link between weight sensations and mental effort, a mediated model was also conceivable (see Figure 1). As stated above, people conceptually associate physical weight with the more abstract concepts of importance and difficulty (cf. Ackerman et al., 2010; Jostmann et al., 2009; Kaspar, 2013a). Hence, it is conceivable that the sensation of weight increases the perceived difficulty of the task and/or its perceived importance. According to the motivational intensity theory (Brehm & Self, 1989), perceived task difficulty improves effort up to the point at which people decide that the potential outcome is not worth the required effort or that a successful performance becomes unlikely. In this sense, the perception of task difficulty should increase effort as anagrams are usually considered as moderately difficult (Boggiano, Flink, Shields, Seelbach, & Barrett, 1993). Similarly, task importance is a motivator of engaged behaviors and it is positively related to effort expenditure (cf. Nie, Lau, & Liau, 2011). Hence, the expected impact of weight on effort may be mediated by perceived difficulty/importance of the task (see Figure 1).

In addition to a weight effect on effort, we also expected an effect on task performance, where previous literature is mixed regarding what could be expected. On the one hand, the sensation of heaviness, compared to lightness, may elicit a higher cognitive elaboration of the test items (cf. Jostmann et al., 2009). This may lead to a more accurate task processing in terms of a more explorative and creative thinking (e.g., Beversdorf et al., 1999; Kumar & Kumari, 1988) as well as persistence (e.g., Eisenberger et al., 1992), reflected by more correctly solved anagrams. However, higher accuracy may be negatively related to task processing speed so that the speed-accuracy tradeoff comes in the focus of interest. Thus, on the other hand, the task performance might be reduced in the heaviness condition due to a slower processing speed in favor of higher accuracy. In this sense, a reduced number of solved anagrams may be a by-product of a changed speed-accuracy tradeoff.

Additionally, two more mechanisms are conceivable that may reduce the task performance. First, heaviness may trigger the impression of a cognitive barrier. Participants may try to bypass this barrier by means of different thinking styles, for example, by a more analytic thinking about task items or, alternatively, by an aimless rumination about the task content. However, rumination as well as analytic thinking (cf. Ansburg & Hill, 2003) counteract creative thinking and, hence, the performance in an anagram task that requires a more playful approach. Second, an increase in perceived task difficulty in the case of a heavy (versus light) weight may increase effort, as depicted in Figure 1, but perceived difficulty sometimes evokes a negative affective state as well (e.g., Paisley & Sparks, 1998), and it is linked to the fear of failure (e.g., Brownlow & Reasinger, 2000). This can lead to avoidance motivation that (again) increases vigilance and analytic thinking but counteracts creative and explorative thinking (Mehta & Zhu, 2009), as also outlined in the regulatory focus theory (Higgins, 1997). Hence, an adverse effect of physical heaviness on task performance could also be motivationally grounded.

In order to capture these different mechanisms (if present), we measured several variables in addition to effort and task performance. Moreover, we intended to scrutinize in which respect task performance may be affected by weight sensations. For this purpose, we investigated if the associative closeness of both the anagrams and the corresponding solutions (i.e., words) to weight (i.e., lightness or heaviness) has an impact on the likelihood of solving anagrams.

### Methods

#### Participants

We tested 45 participants (21 male) with a mean age of 23.23 years (*SD* = 4.91) and no prior experience with anagram tasks. Sample size was selected according to Jostmann et al. (2009) who reported an average sample size of 45 participants across four studies applying a single factor (light vs. heavy) between-subject design. The two groups did not differ in their mean age, *t*(42) = 0.26, *p* = .798, and gender was counterbalanced across conditions (12 females per condition) due to potential gender differences in physical power (cf. Kaspar, Jurisch, & Schneider, 2015). The sample was homogenous regarding their educational background (university students). We recruited all participants at the university campus and then guided them to the laboratory in order to keep the surrounding conditions (i.e., noise, light, and visual input) constant. All participants voluntarily participated in this experiment (as well as in Experiment 2). They were explicitly informed that they will participate in an experiment whose data will only be used for research purposes and that all data will be digitalized and processed anonymously. The two experiments conformed to the Code of Ethics of the German Psychological Association (DGPs).

#### Materials

Participants of the two genders were randomly assigned to either a light or to a heavy clipboard. The weights of the clipboards were 216.5 g for the light one and 813 g for the heavy counterpart. In order to avoid substantial bodily fatigue (the anagram task plus the questionnaires took about 10 min to complete), we selected a weight for the heavy clipboard that was significantly lower than heavy clipboards used in previous studies (e.g., Ackerman et al., 2010: 2014.2 g and 1559.2 g; Jostmann et al., 2009: 1039 g; Kaspar, 2013a: 1690.5 g and 1667.5 g; Kaspar & Krull, 2013: 2026 g). In contrast, the light clipboard was selected following Kaspar (2013a) who used a light clipboard of 216.5 g. This was the minimum weight (tare weight of the clipboard plus questionnaire and task sheets). Other researchers used heavier clipboards for the light condition (Ackerman et al., 2010: 340.2 g and 453.6 g; Jostmann et al., 2009: 657 g; Kaspar & Krull, 2013: 576 g), but then the difference between the two weight conditions would have been too small.

All participants filled out the Questionnaire on Current Motivation (QCM, Rheinberg, Vollmeyer, & Burns, 2001) before the anagram task. This questionnaire measures motivational factors in learning and achievement situations (i.e., fear of failure, probability of success, interest in the task, and challenging potential). These variables enable us to check whether potential group differences in task performance are afflicted by differences in pre-task motivation. Additionally, participants had to predict their performance in the following anagram task on a scale from *very bad performance* to *very good performance* (0-10) as an indicator for optimism.

The anagram task was taken from Kaspar (2013b) and consisted of 25 German nouns with 5-7 letters in mixed order that had to be rearranged (e.g., ‘‘CCTIAT’’ = ‘‘TACTIC’’). Based on the baseline from Kaspar (2013b), we set the time limit to 5 min in order to prevent a ceiling effect. Following Schiffman and Greist-Bousquet (1992), all items were presented on one page so that participants could observe the whole list.

After the anagram task, participants rated the subjective workload in the German version of the NASA Task Load Index (NASA-TLX; Hart & Staveland, 1988) assessing participants’ mental (“How mentally demanding was the task?”), physical (“How physically demanding was the task?”), and temporal demands (“How hurried or rushed was the pace of the task?”). Additionally, it comprises an assessment of one´s performance (“How successful were you in accomplishing what you were asked to do?”), effort (“How hard did you have to work to accomplish your level of performance?”), and current frustration derived from performing the task (“How insecure, discouraged, irritated, stressed, and annoyed were you?”). Each scale ranged from 1 to 20. Following Hamborg, Hülsmann, and Kaspar (2014), we did not apply an individual weighting of the scales (Hart, 2006) because non-weighted scales are highly correlated with the weighted counterparts (Moroney, Biers, & Eggemeier, 1995) and they show a high reliability in the German version of the questionnaire (Pfendler, 1990).

#### Procedure

The whole procedure was completely standardized. Following Jostmann et al. (2009), the experimenter explained at the beginning of the experiment that its purpose was to investigate how the performance in the anagram task is influenced by different body postures. In order to keep body posture constant, participants had to stand and hold the clipboard during the whole procedure. The experimenter handed over the clipboard and told the participants to clasp the clipboard with their nondominant forearm (always left) and hold it in a comfortable position such that its lower part rested on the waist (cf. Jostmann et al., 2009). All participants stood at the same position and looked in the same direction without eye contact with the experimenter who sat 3 m behind them. A coversheet on the clipboard informed the participants about the course of the experiment and the nature of anagrams. After they provided demographic data (i.e., age and sex) and reported on their former experience with anagram tasks, they were asked to fill out the OCM questionnaire and to predict their performance in the following anagram task (optimism rating). Afterwards, subjects had to solve as many anagrams as possible within 5 min. Right after the anagram task, participants rated their subjective workload on the NASA-TLX questionnaire.

#### Data analysis

We report parametrical tests where appropriate, otherwise we report results of corresponding non-parametric tests. Particularly, in the case of inhomogeneous variances, the Welch-test was preferred over the *t*-test for independent samples. Although the *t*-test is considered robust against violations of the normality assumption (e.g., Heeren & D’Agostino, 1987; Sawilowsky & Blair, 1992), we will instead report the result of the Mann-Whitney U-test if tests for normal distribution suggested the rejection of the null hypothesis (Kolmogorov-Smirnov and Shapiro-Wilk) and if skew and kurtosis values additionally revealed a substantial deviation from the normal distribution according to the criteria defined by Miles and Shevlin (2001, p. 74). Appropriate correlation statistics were selected accordingly (Pearson product-moment correlation *r*, Spearman rank correlation *r*_S_, rank-biserial correlation *r*_RB_, or bootstrapping in the context of mediator analyses). Importantly, we generally validated all results of parametric tests by computing the non-parametric counterparts. All results of Experiment 1 (and Experiment 2) remained unchanged—that is, significant results remained significant and non-significant results remained non-significant. We always refer to an uncorrected significance level of .05.

### Results

**Figure 2.**
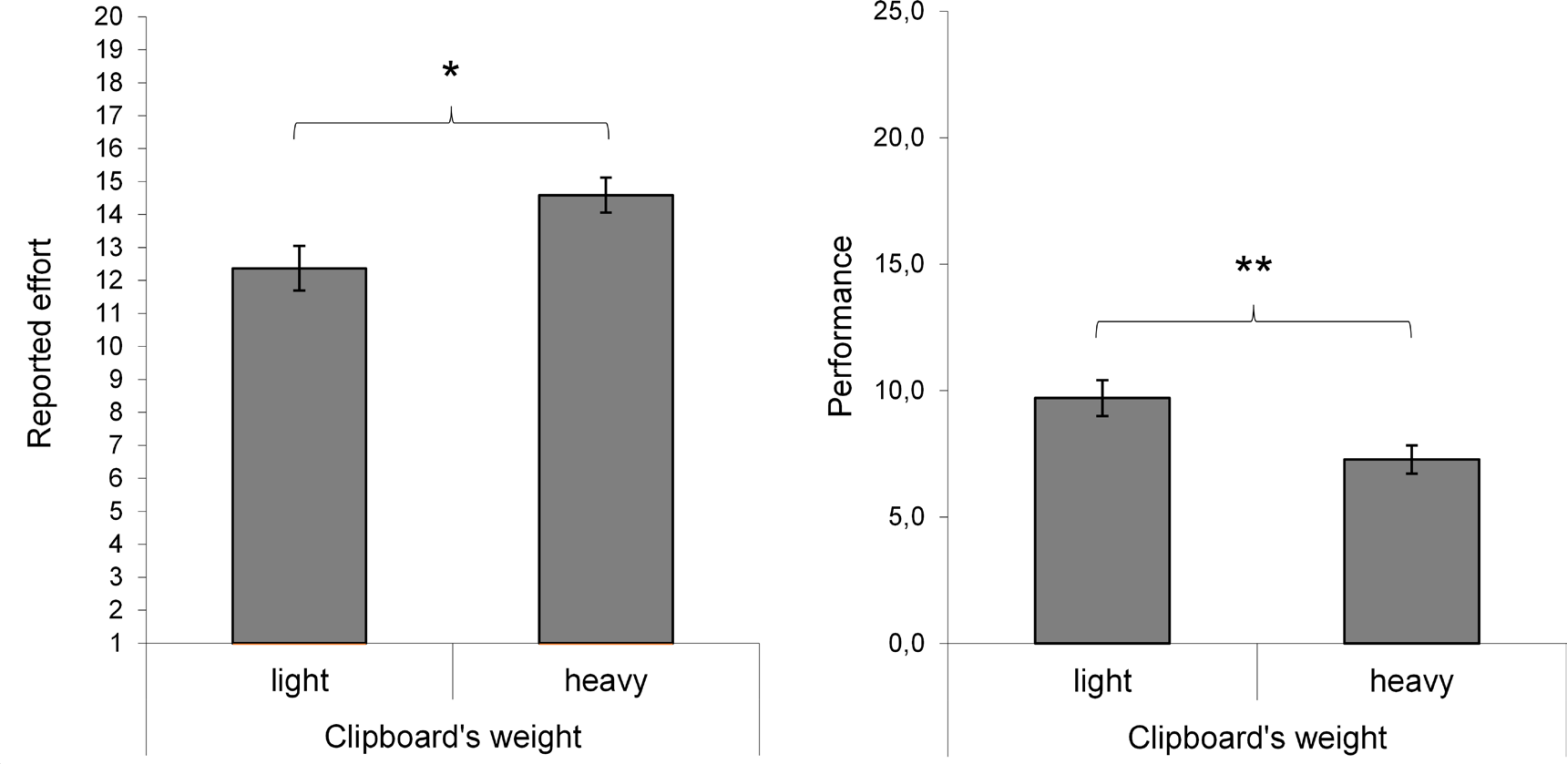
The reported effort after task completion (left side) and task performance in terms of the number of correctly solved anagrams (right side). Error bars represent the standard error of the mean.

As expected, the number of correctly solved anagrams was influenced by the clipboard’s weight, *t*(40.35) = 2.65, *p* = .009, *d* = 0.81. As shown in Figure 2, participants performed better in the light clipboard condition (*M* = 9.74, *SD* = 3.40) compared to the heavy condition (*M* = 7.32, *SD* = 2.50). Moreover, participants in the light clipboard condition (*M* = 12.37, *SD* = 3.25) reported less effort than participants in the heavy clipboard condition (*M* = 14.59, *SD* = 2.49), *t*(43) = -2.57, *p* = .014, *d* = 0.77.

In the next step, we tested the mediation hypothesis according to which the effect of weight on effort is mediated by the perceived task difficulty and/or by task importance. First of all, we computed the mediator values. The reported *fear of failure* and *probability of success* (QCM questionnaire) were averaged and served as an indicator of perceived task difficulty. We also used the mental demands scale of the NASA-TLX as an indicator of perceived task difficulty. The mean across *interest in the task* and *challenging potential* indicated the perceived importance of the task. According to Baron and Kenny (1986) the predictor variable (i.e., dummy-coded clipboard condition; 0 = *light*, 1 = *heavy*) must correlate with the outcome variable (i.e., effort) as well as with the potential mediator (i.e, task difficulty and importance). While the first precondition was fulfilled as already shown by the effect of the clipboard weight on effort, *r* = 0.36, *p* = .014, neither a correlation between the clipboard’s weight and the two measures of task difficulty was found (difficulty according to the QCM scales: *r* = 0.08, *p* = .613; difficulty in terms of the mental demands scale of the NASA-TLX: *r*_RB_ = -0.19, *p* = .790), nor a correlation between the clipboard’s weight and task importance occurred, *r* = -0.21, *p* = .176. Furthermore, we used a more elaborate method by Preacher and Hayes (2008) which directly investigates the mediation hypothesis by testing the difference between the total effect of the predictor variable (i.e., clipboard weight) on the outcome variable (i.e., effort) and the direct effect of the predictor variable on the outcome variable, controlling for several mediators (i.e., task difficulty and importance). This analysis did also not support the mediation model independently of the measure of task difficulty that was included as a mediator, both *effects* ≤ -0.40, *z* ≤ -1.21, *p* ≥ .228. An additional bootstrap analysis for the 95% confidence interval showed that zero was included, indicating no mediation effect. Hence, the effect of weight on perceived effort required by the task was neither mediated by the perceived task difficulty nor by the perceived importance of the task. Instead, the results support the notion of either a direct effect of weight on effort (cf. Jostmann et al., 2009) or, alternatively, that other variables may mediate this effect.

In the next step, we analyzed whether effort mediated the effect of the clipboard’s weight on the task performance, *r* = -0.38, *p* = .010. Because the clipboard’s weight also correlated with the potential mediator effort, *r* = 0.36, *p* = .014, a multiple regression was computed that included the clipboard’s weight and effort as predictor variables (Baron & Kenny, 1986). The two variables jointly explained a significant amount of variance in performance, *R*^2^ = 0.40, *p* = .027, while the clipboard’s weight showed a significant contribution, *t* = -2.23, *p* = .031, in contrast to effort, *t* = -0.79, *p* = .437. Hence, no support for the mediation model was found. This conclusion was also supported by the procedure of Preacher and Hayes (2008) analyzing the indirect effect of weight on performance through effort, effec*t* = -0.28, *z* = -0.77, *p* = .443, 95% CI = -1.12 to 0.22.

To conclude, the sizes of the effect of the clipboard’s weight on effort as well as performance were middle to large (effort: *d* = 0.77; performance: *d* = 0.81), while the two mediation hypotheses (see Figure 1) were not supported by the data.

Importantly, besides the weight effect on perceived effort and the null effect on mental demands (see above) none of the other NASA-TLX scales showed an effect of the clipboard’s weight, all |*t*| ≤ 0.40, *p* ≥ .689, *d* ≤ 0.12. Thus, the sensation of heaviness, in contrast to lightness, did not elicit the impression of higher physical and temporal demands. Also, it did not influence the reported level of frustration as well as the assessed success in accomplishing the task requirements. In addition to these post-task ratings, no significant group differences existed in the reported pre-task optimism, *z* = -0.90, *p* = .369, *d* = 0.31. Consequently, group differences in pre-task optimism did not account for the difference in perceived effort during task completion as well as in the actual performance. Also, there was no weight effect on the four scales of the Questionnaire on Current Motivation (QCM) which were aggregated to assess task importance and difficulty: fear of failure, interest in the task, and challenging potential, all |*t*| ≤ 1.31, *p* ≥ .198, *d* ≤ 0.39, probability of success, *z* = -1.38, *p* = .167, *d* = 0.29.

In the next step, we analyzed whether the effect of the clipboard’s weight on performance derived from a better cognitive access to lightness-related words in the light clipboard condition or, alternatively, a worse access to this cognitive content in the heavy clipboard condition. Thereby, we differentiated between the anagrams as they visually appear, on the one hand, and the corresponding solutions (i.e., words), on the other hand. While heaviness could have hampered the first contact with anagrams that are associated with lightness, heaviness also could have reduced the retrieval of lightness-related words on the level of anagram solutions. For this purpose, a new sample of 47 subjects (39 female) with a mean age of 24.62 years (*SD* =5.97) participated in an online experiment and was randomly assigned either to the list of the anagrams or to the list of the corresponding solutions (i.e. words). They had to judge how much they associate each item with lightness on a 7-point scale ranging from 1 (*not at all*) to 7 (*very*). The items were presented in a random order. We calculated the mean of each item across the respective subjects. Afterwards, these means were used to calculate the mean lightness association score across all items solved by a subject of the main experiment. The resulting score indicated the mean lightness that was associated with the solved anagrams per subject. Two final *t*-tests comparing the light and heavy clipboard condition showed no difference in the lightness association score regarding the anagrams, *t*(43) = -1.05, *p* = .299, *d* = 0.31, as well as regarding the corresponding solution (i.e. words), *t*(43) = 0.12, *p* = .905, *d* = 0.04. Consequently, the effect of the weight sensation on performance did not derive from a hampered cognitive accessibility of certain anagrams in the heavy clipboard condition. That is, the results contradict the possibility that the embodied cue of weight works in terms of semantic priming within specific semantic networks, making the access to specific lexical content more facile.

Finally, we tested whether the negative effect of a heavy clipboard on task performance derived from a more accurate responding. It is conceivable that the heavy clipboard triggered a more elaborate thinking about each anagram (cf. Jostmann et al., 2009), so that participants in the heavy clipboard condition were not as fast as the participants in the light clipboard condition. If so, this should be reflected in a higher accuracy (i.e. less mistakes). Overall, the participants made only few mistakes. We found a marginal trend in the other direction, *z* = -1.80, *p* = .072; that is, participants made slightly more mistakes when they held a heavy clipboard in their hands. Consequently, the effect of perceived heaviness on task performance is not a signature of a detrimental speed-accuracy tradeoff.

### Discussion

In Experiment 1, we found an effect of incidental haptic weight sensations on the number of correctly solved anagrams. Task performance was reduced when participants held a heavy (versus light) clipboard in their hands during task processing. This heaviness effect was accompanied by higher post-task reported effort, but effort did not mediate the weight effect on performance. Moreover, pre-task optimism and motivation (QCM scales) did not differ between the two clipboard groups and thus did not account for the group differences in perceived effort and performance. Also, we did not find evidence that the effect of weight on effort was mediated by the perceived importance and difficulty of the task. Thereby, it was irrelevant whether perceived difficulty was assessed before the task or after the task. There was also no weight effect on reported physical, mental, and temporal demands, as well as on post-task reported frustration. This is important to note as some literature suggested increased negative affect and fear of failure when the perceived difficulty of the task increases (e.g., Brownlow & Reasinger, 2000; Paisley & Sparks, 1998). According to Gendolla and Krüsken (2001), subjective demands should be higher in a negative mood compared to a positive mood. However, we neither found a weight effect on task difficulty, nor on fear of failure, perceived demands, and reported frustration. Thus, the result pattern contradicts the notion that the weight effect on task performance was motivationally grounded.

Furthermore, a more fine-grained analysis of participants’ anagram solutions provided two insights. First, we did not find evidence for a reduced cognitive access to lightness-related items in the heavy clipboard condition. According to the conceptual metaphor theory (Lakoff & Johnsson, 1980), the sensation of heaviness should activate semantically related knowledge. Given this idea, we asked whether activating the concept of heaviness may have reduced the likelihood of solving anagrams which were closely associated with the concept of lightness. The present results did not support this option. Second, the analysis of false anagram solutions (i.e., mistakes) revealed that the reduced performance in the heavy clipboard group is not a signature of a detrimental speed-accuracy tradeoff. As outlined above, one might assume that the sensation of heaviness triggers a more explorative and persistent thinking about each anagram (cf. Jostmann et al., 2009). This may lead to reduced processing speed in favor of a higher accuracy. However, participants made slightly more mistakes when they held a heavy versus a light clipboard in their hands. Thus, the present results do also not support the notion of a reduced processing speed elicited by heaviness sensations. Consequently, the data seem to be more compatible with the view that the sensation of heaviness elicited a cognitive barrier. This perceived barrier could have counteracted fluent creative thinking by stimulating, for example, a more analytic thinking or aimless rumination which participants applied to handle the problem at hand. Although we are not able to specify the nature of this cognitive barrier, it is conceivable that heaviness literally increased the perceived “gravity of the situation” in some form (cf. Ackerman et al., 2010), making fluent task processing more difficult. In fact, some researchers assume that such conceptual metaphors are the basis of embodiment phenomena as they shape the way we think (cf. Lakoff & Johnson, 1980). However, the present data cannot resolve the debate about the role of linguistic metaphors in the context of embodied cognition phenomena. At least we can conclude that the present effect is not a signature of a better cognitive access to lightness-related words in the light clipboard condition. Future studies are necessary to answer whether bodily sensations may work as a semantic prime that, in turn, affects higher cognitive processes.

Finally, we once more want to point out that the perceived physical and mental demands were not affected by the weight sensation. Both measures did also not correlate with effort, both *r* 0.21, *p* 0.17. However, we cannot exclude the possibility that the effort scale also captures some unspecific aspects of mental or physical fatigue, although we tried to avoid physical exhaustion by using a heavy clipboard that was lighter than those used in previous studies. Hence, it is possible that the impression of a cognitive barrier elicited by a heavy clipboard may be (at least partially) determined by some kind of fatigue. However, the fact that we did not find a weight effect on the reported physical demands means that we may conclude that the weight treatment did not elicit physical exhaustion substantial enough to completely explain the lowered task performance in the heavy clipboard condition. All in all, the present experiment shed first light on weight effects on cognitive performance, but it also left some open questions. Consequently, we conducted a second experiment to further investigate the mechanism behind such weight effects.

## Experiment 2

In Experiment 2, we scrutinized whether the weight sensation has actually no influence on participants’ speed-accuracy tradeoff that could account for the effects on performance. Heaviness might trigger a more elaborate thinking about an issue (Jostmann et al., 2009). Thus, heaviness might slow down the processing speed in favor of higher accuracy. Experiment 1 was limited in this respect so that no final conclusion could be drawn. On the one hand, the task processing time was set constant for all participants, reducing the degrees of freedom for a self-imposed processing speed. On the other hand, only few mistakes had been made overall so that the accuracy analysis could be biased by some few random events. Hence, a task was required that allows both a self-imposed speed-accuracy tradeoff as well as a simple response heuristic. The latter option was necessary to test the alternative hypothesis that heaviness elicits the impression of a cognitive barrier participants try to bypass by applying a specific processing style. With respect to Experiment 1, we assumed that the heavy clipboard may have triggered a more analytic thinking style or aimless rumination that counteracted what was required by the anagram task: creative thinking. However, we were not able to make this processing style visible so that further alternative explanations (in addition to those excluded by the data of Experiment 1) are possible. Consequently, we needed a task that could make a specific processing style or response heuristic visible.

To meet these requirements, we constructed a two-alternative forced-choice task (left versus right) with a new kind of visual stimulus (see methods section). Thereby, the task was characterized by considerable uncertainty due to the absence of substantial diagnostic information regarding the right choice so that embodied information should be significantly incorporated in the task processing style (cf. Binder & Desai, 2011; Kaspar, 2013a; Landau, Meier, & Keefer, 2010). If heaviness actually triggers a more elaborate thinking about task items, longer task completion times in the heavy clipboard condition should coincide with higher hit rates. Alternatively, if heaviness elicits the impression of a cognitive barrier, participants should apply a simple response heuristic to bypass this barrier: in accordance with the body-specific hypothesis (Casasanto, 2009), right handers usually allocate positive affect to the rightward body space, while this association seems to be an effect of repeated successful motor actions. In fact, when the right hand of right-handers is temporally handicapped, they show preferences for the left side (Casasanto & Chrysikou, 2011). Thus, we assumed that the sensation of heaviness, if it actually triggers the impression of a cognitive barrier, should lead to a simple “right is the better choice” heuristic in order to maintain a fluent task processing. In this case, we also expected no weight effect on effort as the primary purpose of this response heuristic should be the avoidance of increased effort.

### Methods

#### Participants

We assessed the required sample size by means of GPower (Faul, Erdfelder, Lang, & Buchner, 2007) on the basis of the effect sizes found in Experiment 1 (effort: *d* = 0.77; performance: *d* = 0.81). Given the smaller effect size of 0.77, a power of .90, a significance level of .05, and a two-tailed hypothesis testing, we got a target sample size of n = 37 for each of the two groups (heavy vs. light). Correspondingly, we analyzed the data of 77 participants (38 vs. 39) while one participant has been excluded prior to the analysis due to several missing data. The two groups of university students did not differ in their mean age, *t*(42) = 0.50, *p* = .617, and gender was again counterbalanced across conditions (19 males per condition). Following Casasanto (2009), we only tested participants who reported to be right-handers and verified this report by the observed writing hand.

#### Materials

**Figure 3.**
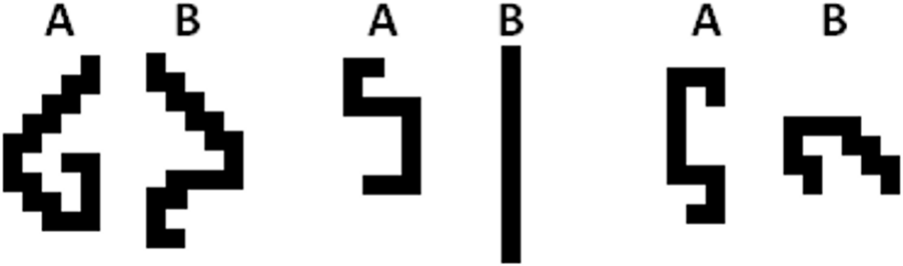
Three examples of the two-alternative forced-choice task used in Experiment 2. Participants had to indicate for each pair which of the snakes is longer by marking A or B.

As in Experiment 1, we applied the QCM questionnaire before the task and the NASA-TLX after the task. However, in contrast to the anagrams of Experiment 1, we created a new set of stimuli being suitable for a two-alternative forced-choice task. Because the stimuli had to be unfamiliar to all participants and because they should nevertheless be easy to grasp, we created visual stimuli that were inspired by the old-fashioned video game “Snake” (see Figure 3). For each pair of snakes, participants had to decide which one is longer by marking A or B with a cross. Hence, a higher accuracy can be achieved by a more accurate visual inspection of the snakes. Overall, participants had to process 24 pairs of snakes, whereby in half of the trials the longer snake was depicted on the right side. All pairs were depicted on one page that was divided into four columns and six rows. The order of snake pairs was identical for all participants.

#### Procedure

The procedure was identical to that in Experiment 1, with the exceptions noted. After the experimenter had reached a passer-by in the main hall of the university, she asked her/him to participate in an experiment on the effect of body posture on cognitive performance (same cover story as in Experiment 1). She highlighted that the three best participants will win 12, 10, or 8 Euros, respectively. The monetary incentive was used to elicit high motivation in all participants. After a passer-by had agreed to participate, the experimenter led him or her to the laboratory. The participant was randomly assigned to the light or the heavy clipboard. The experimenter explained the procedure and handed over the corresponding clipboard. The cover page described the snake task in detail and, once more, highlighted the monetary gain for the best three performers, for which completion time and accuracy were considered. Afterwards, participants filled out the QCM questionnaire. Then, on the next page, the snake task was presented. Participants performed the task without a time limit, but completion time was recorded by the experimenter. In the end, they filled out the NASA-TLX questionnaire. After all participants had been tested, the best three of them were identified by means of the hit rate/completion time ratio. They were contacted via e-mail and were paid the promised monetary gain.

### Results

**Figure 4.**
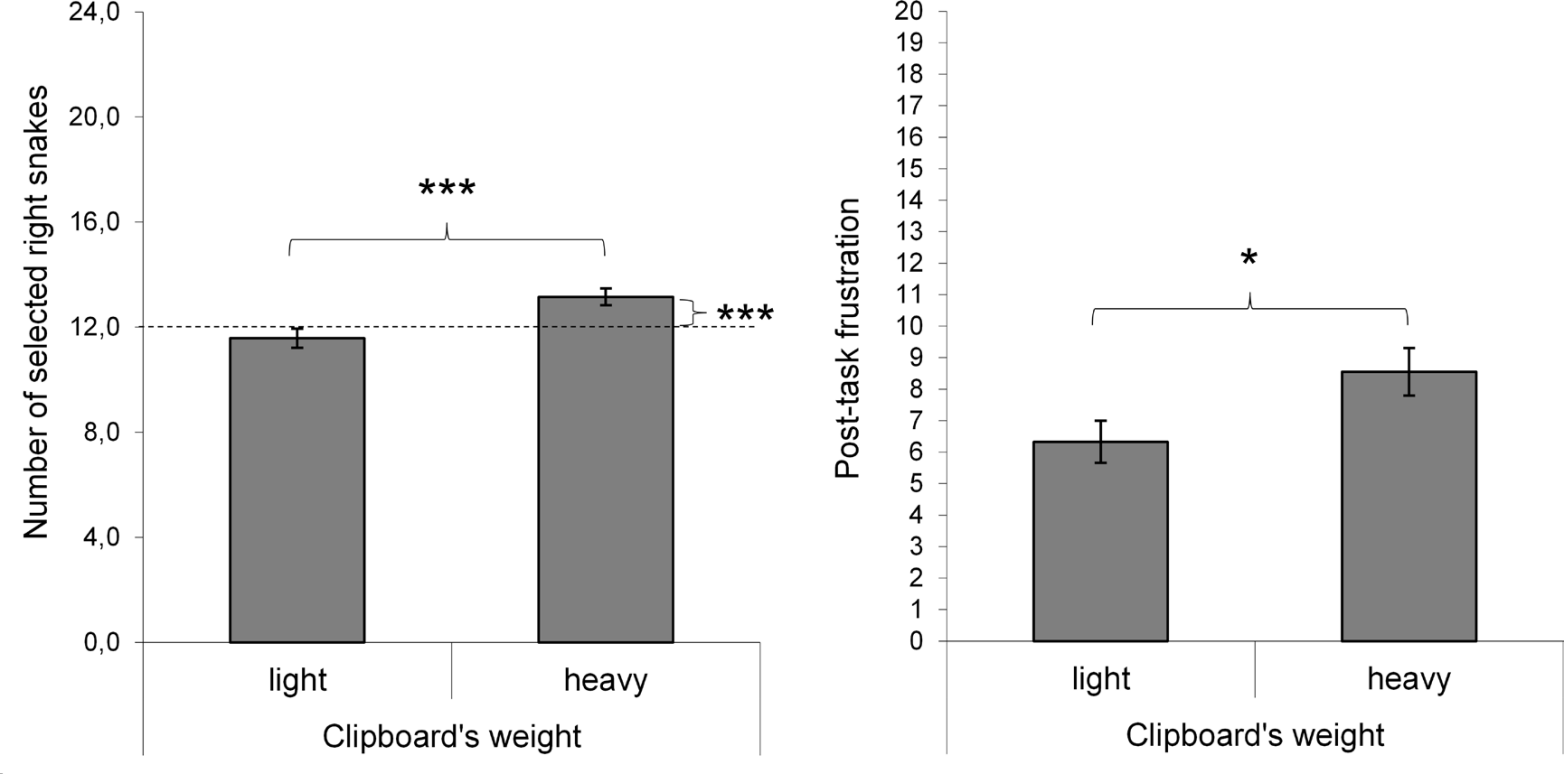
The number of selected right snakes (left side) and post-task reported frustration (right side). The dotted line in the left diagram indicates 50% of trials. Error bars represent the standard error of the mean.

Task completion time and the number of correctly identified snakes correlated slightly positively, *p*_S_ = 0.20, *p* = .083. However, and contrary to the assumption that a heavy clipboard triggers a more elaborate item exploration and, thus, slows down task processing in favor of more accurate responses, we neither found an effect of the clipboard’s weight on task completion time, *z* = -0.72, *p* = .473, *d* = 0.09, nor on the number of correctly identified snakes, *t*(75) = 0.23, *p* = .818, *d* = 0.05. The mean hit rate (*M* =15.86, *SD* = 2.38) was significantly above the chance level of 50%, *t*(76) = 14.24, *p* < .001, *d* = 1.62. But, in accordance with the notion of a simple response heuristic in the case of a sensed heaviness, participants in the heavy clipboard condition selected the right snake of a pair more often than participants in the light clipboard condition, *t*(75) = -3.30, *p* = .001, *d* = 0.75. As shown in Figure 4, in the light clipboard condition the number of selected left and right snakes did not differ from 12 (i.e., 50% of trials), both |t| = 1.18, *p* = .246, *d* = 0.19. In contrast, in the heavy clipboard condition the number of selected right snakes (54.81%) was above 50% (*M* =13.15, *SD* =1.98) and the number of left snakes (45.19%) below 50% (*M* =10.85, *SD* =1.98), both |t| = 3.64, *p* = .001, *d* = 0.58. Hence, the two groups differed significantly from each other, *t*(75) = 3.30, *p* = .001, *d* = 0.75. This contrast is also reflected in an interaction, *F*(2, 75) = 10.90, *p* = .001, η_p_^2^ = .13, when computing a 2 × 2 (clipboard condition × side of selected snakes) mixed-measures ANOVA. We did not find an effect of the side, *F*(2, 75) = 2.36, *p* = .129, η_p_^2^ = .03. No effect for the clipboard condition was computed because it is a constant instead of a variable (i.e., a value of 24 for all participants). Moreover, the right side bias in the heavy clipboard condition coincided with an increased post-task reported frustration in the heavy clipboard condition (*M* =8.55, *SD* =4.74) compared to the light condition (*M* =6.33, *SD* =4.12), *z* = -2.06, *p* = .039, *d* = 0.50. Consequently, although the clipboard’s weight did not affect task processing speed and accuracy, it elicited a right side bias in the two-alternative forced-choice task. Hence, this response tendency did not thwart an accurate item processing. Apparently, the correctly solved items substantially varied across participants so that the right side bias did not affect task performance.

In contrast to Experiment 1, we found no effect of weight on post-task reported effort, *t*(75) = -0.88, *p* = .382, *d* = 0.20. In accordance with Experiment 1, we found neither a weight effect on the other scales of the NASA-TLX questionnaire, all |*z*| ≤ 1.19, *p* ≥ .236, *d* ≤ 0.27, nor on the pre-task optimism rating, *t*(75) = 0.19, *p* = .848, *d* = 0.04. Also, we found no weight effect on the assessed task difficulty and task importance operationalized by aggregating the scales of the QCM questionnaire (see Experiment 1), both |*t*| ≤ 0.66, *p* ≥ .509, *d* ≤ 0.15. As we did not find an effect of the clipboard’s weight on task performance, completion time, and effort, we abstain from reporting the results of corresponding mediation analyses due to insignificant results in all cases. However, we exploratorily tested whether the effect of weight on the number of selected right snakes was mediated by frustration. Both measures significantly correlated with weight as reflected by the above mentioned main effects. We computed a multiple regression analysis that included the clipboard’s weight and frustration as predictor variables, and the number of selected right snakes as criterion (Baron & Kenny, 1986). However, we found no mediation effect. The two predictor variables jointly explained a significant amount of variance, *R*^2^ = 0.36, *p* = .006, where the clipboard’s weight showed a significant contribution, *t* = 3.30, *p* = .001, in contrast to frustration, *t* = -0.46, *p* = .644. This conclusion was also reached by the procedure of Preacher and Hayes (2008) analyzing the indirect effect of weight through frustration, *effect* = -0.06, *z* = -0.46, *p* = .646, 95% CI = -0.47 to 0.16.

### Discussion

In contrast to Experiment 1, we found no effect of haptic weight sensations on task performance, but we were able to uncover a specific task processing style. The sensation of heaviness, compared to lightness, did not affect participants’ speed-accuracy tradeoff, contradicting the assumption that heaviness may elicit a more elaborate thinking about an issue and, hence, slow down task processing speed in favor of higher accuracy. This assumption was proposed by Jostmann et al. (2009) who found a higher consistency between related judgments as an indicator for a more in-depth elaboration of test items. In contrast, the present data suggest that a heavy clipboard triggered a specific response heuristic in terms of a simple “right is the better choice” rule. In several studies, right handers were found to associate the right side of their body space with higher positive valence (e.g., Casasanto, 2009) and to search for a target on the right side in a T-maze task (Scharine & McBeath, 2002). Importantly, this right side bias seems to be grounded on the experience of a more fluent and successful interaction with the environment when using the dominant hand, because the right side bias can be flipped into a left side bias by handicapping the right hand in a motor coordination task (Casasanto & Chrysikou, 2011). Accordingly, and suggested by the findings of Experiment 1, we considered the possibility that the sensation of heaviness may elicit the impression of a cognitive barrier which participants would try to bypass by choosing the right response option more often in order to maintain fluent task processing and to avoid additionally increased effort. The present data supported this second option instead of a modulation of the speed-accuracy tradeoff. It has to be stressed that this response heuristic did not lead to a shorter task completion time, indicating that participants in the heavy clipboard condition were trying to solve the items, but that they were influenced by the clipboard’s weight along the way rather than giving up and, therefore, simply choosing the right response option more often. In fact, the results of Experiment 1 also did not support the notion of a change in participants’ speed-accuracy tradeoff. However, the anagram task of Experiment 1 did not allow applying a similarly simple response heuristic to cope with the influence of weight. Instead, the sensation of heaviness may have reduced performance by eliciting a more analytic thinking style or aimless rumination about the task content (which was not observable, however).

However, we want to emphasize that, at the present moment, we can only speculate about the functional nature or mechanism of what we called a “cognitive barrier”. Future research is necessary to further scrutinize this crucial point. Nonetheless, the present data provide some additional hints. First, in Experiment 2, we found no effect of weight on post-task reported effort. Given that the weight manipulation did not affect task performance (i.e., the number of correctly identified snakes) and task completion time, there was no need to feel more exhausted when holding a heavy versus a light clipboard. Instead, the weight-related change in participants’ response pattern (i.e., more right than left snakes in the heavy clipboard condition) apparently counteracted the potential increase in effort. This is the important difference between Experiments 1 and 2, highlighting that weight effects seem to depend on the type of the cognitive task. Also, weight did not affect the post-task reported mental demands. However, and in contrast to Experiment 1, we found that the right side bias introduced by a heavy clipboard was accompanied by a higher post-task frustration. This may reflect that the participants actually experienced some cognitive barriers during task processing. Perhaps participants in the heavy clipboard condition were less satisfied with the simple response heuristic they applied to handle the cognitive barrier.

Finally, it has to be mentioned that, as in Experiment 1, weight did not affect the pre-task motivation and optimism. Also, weight again had neither an effect on the post-task assessed success in accomplishing what the task required, nor did weight influence the assessed temporal demands of the task. Indeed, the number of correctly identified snakes and the task completion time were identical in both groups. Again, we found no effect of physical weight on the reported physical demands of the task.

## General discussion

Previous studies provided strong evidence that the incidental sensation of physical weight affects the evaluation of issues and objects on dimensions that are conceptually related to weight. The research question of the present work was whether the sensation of weight also affects one’s performance in cognitive tasks. Our results support this assumption, showing an effect that is not unspecific but task-dependent. In Experiment 1, the performance in an anagram task was reduced when participants sensed heaviness compared to lightness. In Experiment 2, a heavy weight affected the task processing style in a two-alternative force-choice task. Participants tended towards a simple response heuristic in terms of a right side bias while their self-imposed speed-accuracy tradeoff and the overall task performance did not change.

Future studies are necessary to scrutinize which variables could mediate such embodiment effects as mediation models have been widely neglected so far in embodied cognition research. The present experiments aimed at providing insights into potential mediation mechanisms, but we found no evidence for that. Perhaps weight indeed affected task performance (Experiment 1) and task processing style (Experiment 2) in a direct manner. We measured several variables that were assumed to mediate these effects as well as several control variables. However, only effort (Experiment 1) and frustration (Experiment 2) were sensitive to the weight treatment, but both variables did not mediate the effect of weight. It might be that this result is due to the fact that these variables were measured after task processing and, hence, perhaps did not reflect states of effort and frustration experienced during task processing. Perhaps a continuous measure of effort during task processing, such as the pupil diameter or the skin conductance level, would be more suitable to capture mediation effects. In this context, we also want to point out that the phenomenology of weight sensations has been widely neglected so far in the embodiment literature (cf. Meier et al., 2012). A bulk of studies reported effects of weight on diverse psychological dimensions. However, at this moment we only can speculate about how the weight itself is perceived. Recently, Kaspar et al. (2015) speculated whether different weight sensations may trigger specific affective responses. This might be a fruitful starting point for future research because it is a crucial point to uncover what kind of mechanism (e.g., a cognitive barrier, shifting one’s attention to specific information, or a kind of cognitive or physical fatigue) mediates weight-related embodiment phenomena.

Nonetheless, a direct link between weight sensations and cognitive performance is actually conceivable. Wilson (2002) outlined that some parts of cognition are externalized and body-based. Thus, it is possible that embodied information directly influences cognitive processing such as problem solving without the necessity to be translated into more abstract cognitive concepts (e.g., difficulty, importance, or perceived effort) to be cognitively effective. In fact, this direct pathway is completely compatible with the core understanding of how embodied cognition may work.

The effect sizes found were middle to large (Cohen’s *d* between 0.50 and 0.81) and are in line with previous studies comparing the effect of light versus heavy clipboards (e.g., Ackerman et al., 2010; Jostmann et al., 2009; Kaspar, 2013a; Kaspar et al., 2015; Kaspar & Krull, 2013). However, Kaufmann and Allen (2014) found only null effects when investigating the impact of backpacks differing in weight on several judgments that were unrelated to the weight manipulation. Apparently, bodily sensations have no effect when they are irrelevant. To the best of our knowledge, in all previous cases of significant effects the weight of an object (e.g., a book, a clipboard, or a backpack) was somehow related to the task. In studies with clipboards, for example, subjects always judged things (e.g., social issues or persons) that were presented on top of the clipboard. Consequently, there was a direct link between the clipboard and the task—at least a spatial and temporal link. This spatiotemporal relationship between the task and the weight manipulation appears to be crucial. If the sensation of physical weight is completely unrelated to the task, the weight sensation may not carry over to the task. Hence, weight-related embodiment effects seem to be context-sensitive and do not generalize to all situations.

Moreover, it is conceivable that physical weight can have a contrast effect on a cognitive dimension. In fact, Kaspar (2013a, Study 5) found that pharmaceutical drugs presented via written vignettes and images on a heavy or a light clipboard were rated as more effective in the light clipboard condition. The author discussed this unexpected result in terms of a negative priming effect. Similarly, it might be that participants who are primed for weight-related concepts (e.g., seriousness) show contrast effects if the task content does not fit to the participants’ expectations. For example, the sensation of heaviness might trigger the concept of seriousness that, however, may lead to reduced ratings of the seriousness of diseases when the diseases to be judged are very mild in general (cf. Kaspar, 2013a).

Furthermore, one might consider the possibility that afferent signals are not needed to produce weight-related effects. It may be sufficient to activate corresponding motor commands or to prime the concept of heaviness in order to induce such effects. In fact, studies on the relationship between physical cleansing and moral judgments showed that actual cleansing is not necessary to change moral judgments (Zhong, Strejcek, & Sivanathan, 2010). According to the moral-purity metaphor, the concept of physical purity serves as a scaffold for the abstract concept of moral purity (cf. Lakoff & Johnson, 1980; Williams et al., 2009). Accordingly, Zhong et al. (2010) found that activating the concept of physical purity by a visualization task led to harsher moral judgments. However, this priming effect was smaller compared to real hand cleansing perhaps because the actual sensorimotor activity is a more powerful prime or, alternatively, because real hand cleansing makes it easier to ascribe the state of physical purity to oneself and, hence, renders the embodied information more influential. It is conceivable that a similarly attenuated but still significant effect of priming could be observed with respect to physical weight. This is an important aspect that would help to better assess the extent to which embodied cognition depends on one’s physical interaction with the environment. However, it has to be noted that such a priming effect is not necessarily of an abstract semantic origin. It might be that a specific prime stimulates the cognitive simulation of interacting with weighty objects. Barsalou (2008) stated that such “simulation is the reenactment of perceptual, motor, and introspective states acquired during experience with the world, body, and mind” (p. 618). In this sense, a prime might activate the sensorimotor experiences related to the bodily interaction with physical objects instead of activating abstract semantic knowledge related to weight. Hence, conceptual metaphors (cf. Lakoff & Johnson, 1980; Lee & Schwarz, 2014) may not be the central mechanism. Studies that would decidedly focus on such priming effects in the absence of actual bodily sensations would be desirable for a more complete picture of the mechanisms.

Also, it might be possible that the weight difference between a light and a heavy condition directly determines the effect size that can be found on the level of the dependent variable. However, although the heavy clipboard in the present studies was lighter than in previous studies, it produced remarkable effects. Indeed, across all previous studies no consistency existed regarding the selected weights. It might be a useful contribution to the literature if the impact of varying weight differences on specific psychological dimensions would be systematically investigated.

The present results also have practical implications. Given that the performance in cognitive tasks can be linked to bodily states, it might be useful in work settings to uncover potential barriers for cognitive performance that derive from specific bodily actions. This could be the case, for example, when tasks require both physical effort as well as cognitive flexibility. This aspect is also of interest regarding human-computer interfaces when mobile devices such as tablets and smartphones provide specific weight sensations. In this sense, considering the impact of weight on product evaluations might increase the effectiveness of classical usability tests (cf. Kaspar, Hamborg, Sackmann, & Hesselmann, 2010). Additionally, specific body-related experiences may be a new avenue for training and intervention aiming at an improvement of cognitive abilities. However, future research is necessary to examine the generalizability of the present results to other cognitive tasks. Perhaps some kind of task also benefits from the sensation of heaviness.

Additionally, we want to emphasize that the two present experiments were associated with very different task requirements. While the anagram task of Experiment 1 required creative thinking and persistence, in Experiment 2 a two-alternative forced-choice task related to other S-R mapping tasks was performed that required an accurate visual analysis of the stimulus being similar to the classical visual discrimination task by Solomon E. Asch (1956) who used lines of different length. Although we assumed that a cognitive barrier was the driving force behind the effects in both experiments, it is also possible that this barrier differed between tasks.

Finally, we want to make a methodological remark: all previous studies as well as the present work on weight effects used between-subjects designs. Although a randomized assignment of participants to weight conditions is common and aims towards an equal distribution of all potential confounds, sometimes sampling errors might occur nonetheless. Thus, replication studies are necessary in this research area. One should maybe also consider using within-subject designs, but the difference in weight between conditions can be striking when measurement time points are very close to each other. This might raise suspicion. Additionally, repeated measures suffer from potential carry-over effects or varying reliabilities of instruments. However, a clever research design using repeated measures and avoiding common problems of within-subject designs might contribute significantly to the field.

To conclude, the present experiments provided first evidence that the incidental sensation of heaviness can affect task performance, task processing style, but also effort and frustration reported post task. These results complement previous research showing that weight sensations affect different kinds of social judgments and object evaluations. Hence, the present results call for more attention to cognitive processes beyond judgment formation in the field of embodied cognition. Based on the novelty of present results many new research questions arise. Overall, the present findings are a promising starting point for future research that should further expand the scope to outcome measures beyond judgments. Moreover, in order to develop elaborate models of embodied cognitive processes, it is important to scrutinize boundary conditions of corresponding phenomena and the role of potential mediators. This will help to deepen our understanding of the mechanisms behind the fascinating interplay between basal bodily sensations and higher cognitive processes.
